# A multi-center study evaluating the correlation between meibomian gland dysfunction and depressive symptoms

**DOI:** 10.1038/s41598-021-04167-x

**Published:** 2022-01-10

**Authors:** Zhenyu Wei, Junqi Liang, Kai Cao, Leying Wang, Christophe Baudouin, Antoine Labbé, Qingfeng Liang

**Affiliations:** 1 Beijing Institute of Ophthalmology, Beijing Tongren Eye Center, Beijing Tongren Hospital, Capital Medical University, Beijing Key Laboratory of Ophthalmology and Visual Sciences, Beijing, 100005 China; 2 School of Medicine, University of Central Lancashire, Preston, UK; 3 Quinze-Vingts National Ophthalmology Hospital, IHU FOReSIGHT, Paris and Versailles Saint-Quentin-en-Yvelines University, Versailles, France; 4 Institut de la Vision, IHU FOReSIGHT, Sorbonne Université, INSERM, CNRS, 17 rue Moreau, 75012 Paris, France

**Keywords:** Eye diseases, Psychiatric disorders, Risk factors

## Abstract

Increased prevalence of depression has been found in patients with meibomian gland dysfunction (MGD); however, specific conditions of patients suffered from MGD and depression remains unclear. Our aim was to investigate the prevalence of depression in patients with MGD and analyze the risk factors of depression in MGD patients. In this multi-center cross-sectional study, we enrolled 830 MGD patients and 114 normal controls. Demographic information was collected by designed questionnaires about lifestyle habits, systemic and ocular medical history. Ophthalmic examinations were performed in a formal order. Depression symptoms were evaluated with a questionnaire survey using a modified self-rating depression scale (M-SDS). The correlations between the M-SDS score and demographic and clinical information were analyzed. The prevalence of hyperlipidemia and obstructive sleep apnea–hypopnea syndrome (OSAHS) was higher in the MGD group. There were 78 cases (9.4%) with depressive symptoms in the MGD group whereas there were 4 cases (3.5%) in controls. Similarly, M-SDS was higher in the MGD group. The characteristics of depression in the MGD group included: crying spells, sleep disturbance and depressed appetite. Some systemic diseases (OSAHS, allergy, skin disease) and the use of contact lenses was correlated with an increased risk of depressive symptoms in MGD patients.

## Introduction

Meibomian gland dysfunction (MGD) is a chronic, diffuse abnormality of the meibomian gland, which can induce the alteration of tear film lipids, a decrease of tear film stability and symptoms of eye irritation. As a leading cause of dry eye disease (DED), MGD becomes a major public ocular health problem, which prevalence rate varies from 3.5% to almost 70%^1, 2^. Its occurrence is always related to gender, age, and race, and a strikingly higher prevalence appeared in the Asia population (46.2–69.3%)^3–5^. In recent years, with the change of social lifestyle and the increase of video display terminal (VDT) viewing, the number of MGD patients has increased significantly^6^.

Clinically, persistent ocular surface discomfort from MGD, including ocular irritation, dryness, burning sensation, and blurring of vision, could always produce a negative impact on life quality. Notably, these symptoms from MGD may be overlap or very similar to those reported in DED patients^7^. Some studies reported the relationship between DED and psychiatric alteration. Especially, Inomata et al.^5^ used Dry Eye Rhythm to collect real-world data and found that severe DE symptoms were correlated with an increased risk of depressive symptoms. This relationship was also confirmed by a meta-analysis^8^, which found a higher depression prevalence in DED patients (29%) than that in controls.

Although MGD becomes a leading cause of DED, few studies attempted to explore the relationship between mental health and MGD. Especially, a potential concern is that chronic symptoms from MGD could induce a negative impact on mental health and the treatment effect of MGD cannot be completely satisfactory^9^. Therefore, it is important to undertake a large-sample study to investigate the MGD patients' mental alteration to make clear the relationship between MGD and depression. With this multi-center epidemic research, we hope to find the risk factors of depressive symptoms in MGD cases and achieve the purpose of early detection and intervention of depression in patients with MGD.

## Results

### Patient characteristics

A total of 944 participants were included: 830 patients with MGD and 114 control subjects. The mean age was 42.6 ± 13.2 years (range 18–87 years) in the MGD group and 40.3 ± 14.6 years (range 18–76 years) in the control group. The number of women in the MGD group and control group was 540 (65.0%) and 84 (73.7%), respectively. There was no significant difference in age (*P* = 0.057), gender (*P* = 0.068) and living area (urban or rural, *P* = 0.188) between these two groups.

The demographic data and basic medical history of MGD patients and normal controls are summarized in Table 1. The survey of systemic diseases showed that the MGD group had higher prevalence of hyperlipidemia (8.8% vs. 0.8%, *P* = 0.003) and OSAHS (obstructive sleep apnea–hypopnea syndrome) (15.9% vs. 7.9%, *P* = 0.024) compared with controls. There was no significant difference for other disease prevalence (hypertension, diabetes mellitus, coronary heart disease, cerebral infarction, allergy, skin disease) within the two groups. Similarly, there was no difference in ocular medical history between the two groups (diabetic retinopathy, glaucoma, cataract, ametropia and contact lens wear).

**Table 1:** Characteristics of MGD patients and normal controls.

Parameters	Normal controls (n = 114)	MGD group (n = 830)	*P* value
**Age**	40.3 ± 14.6	42.6 ± 13.2	0.057
**Gender [n (%)]**			
Male	30 (26.3%)	290 (34.9%)	0.068
Female	84 (73.7%)	540 (65.1%)	
**Residence [n (%)]**			
Urban	107 (94.0%)	747 (90.0%)	0.188
Rural	7 (6.0%)	83 (10.0%)	
**Systemic diseases [n (%)]**			
Hypertension	10 (8.8%)	73 (8.8%)	0.993
Hyperlipidemia	1 (0.8%)	73 (8.8%)	0.003*
Diabetes mellitus	3 (2.6%)	25 (3.0%)	1.000
Coronary heart disease	0 (0.0%)	9 (1.1%)	0.610
Cerebral infarction	0 (0.0%)	5 (0.6%)	1.000
OSAHS	9 (7.9%)	132 (15.9%)	0.024*
Allergy	10 (8.8%)	79 (9.5%)	0.798
Skin disease	5 (4.82%)	43 (5.18%)	0.717
**Ocular diseases [n (%)]**			
Diabetic retinopathy	1 (0.8%)	3 (0.3%)	0.403
Glaucoma	0 (0.0%)	1 (0.1%)	1.000
Cataract	8 (7.0%)	48 (5.8%)	0.601
Ametropia	92 (63.9%)	500 (60.2%)	0.408
Contact lens wear	4 (3.5%)	27 (3.25%)	0.782

*OSAHS* obstructive sleep apnea–hypopnea syndrome.

*P < 0.05 was considered statistically significant.

In the MGD group, 86 (10.4%) cases smoked and their smoking index was 159.3 ± 26.8, which were higher than that in normal controls (5.3%, 73.2 ± 12.1; all *P* < 0.05). In addition, one-fifth of MGD cases always drank alcohol, higher than the control group (*P* = 0.001). Although the duration of sport and walking every day in these two groups were no significant different, the reading time in MGD (60% cases, almost every day), is longer than that in the control group (48.2%, almost every day, *P* = 0.029, Table 2).

**Table 2:** Lifestyle habits of MGD patients and normal controls.

Parameters	Normal controls (n = 114)	MGD group (n = 830)	*P*-value
**Smoking**			
Number of smokers [n (%)]	6 (5.3%)	86 (10.4%)	0.041*
Number of non-smokers [n (%)]	108 (94.7%)	744 (89.6%)	
Smoking index of smokers^a^	73.2 ± 12.1	159.3 ± 26.8	0.032*
**Drinking**			0.745
Number of Drinkers	9 (7.9%)	172 (20.8%)	0.001*
Number of Non-Drinkers	105 (92.1%)	658 (79.2%)	
Alcohol drinking index of drinkers^a^	283.5 ± 22.3	325.2 ± 45.3	0.496
**Reading [n (%)]**			
0 (never)	0 (0.0%)	32 (3.9%)	0.029
1 (almost never)	22 (19.3%)	100 (12.1%)	
2 (1-2 day/week)	14 (12.3%)	87 (10.5%)	
3 (3-5 day/week)	23 (20.2%)	113 (13.6%)	
4 (almost every day)	55 (48.2%)	498 (60.0%)	
**Sports (h/week)**			
Low intensity	3.6 ± 2.2	2.6 ± 1.4	0.092
Medium intensity	2.0 ± 1.2	2.3 ± 2.0	0.404
High intensity	1.3 ± 1.1	1.0 ± 1.2	0.903
**Walks (> 30 min) [n (%)]**			
0 (never)	74 (65.0%)	521 (62.7%)	0.280
1 (2-3 days/week)	11 (9.6%)	125 (15.6%)	
2 (4-5 days/week)	26 (22.8%)	144 (17.3%)	
3 (6-7 days/week)	3 (2.6%)	40 (4.8%)	
Inactive time (h/day)	5.6 (3.0–7.5)	4.7 (3.6–6.2)	0.442

*P < 0.05 was considered statistically significant.

^a^Smoking and drinking index were calculated as follows: Smoking index = (number of cigarettes consumed per day) × (years of smoking); Alcohol drinking index = (Gram of Liquor consumed per day) × (years of drinking).

### Clinical manifestations

Ocular examination revealed no significant difference between MGD patients and normal controls in BCVA and IOP (*P* = 0.451*,* 0.078). Compared with normal controls, MGD patients had higher OSDI score (26.0 ± 15.1 vs. 6.5 ± 4.8, *P* < 0.001), shorter TBUT (4.1 ± 2.3 s vs. 7.3 ± 4.2 s, *P* = 0.002), lower LLT (51.7 ± 24.9 nm vs. 67.9 ± 21.9 nm, *P* = 0.004) and a higher Oxford scale (0.7 ± 1.2 vs. 0.2 ± 0.6, *P* = 0.012). In the MGD group, the score of the lid margin and the loss rate of meibomian glands were higher than normal controls (2.9 ± 0.8 vs.1.2 ± 0.7, 34.5 ± 12.2 vs. 11.5 ± 11.9; all *P* = 0.002). Meanwhile, the higher score of MG expressibility (1.7 ± 0.1 vs. 0.6 ± 0.1, *P* = 0.001) and Meibum (2.2 ± 0.2 vs. 0.5 ± 0.2, *P* = 0.010) were also detected in the MGD group, comparing with normal controls (Table 3).

**Table 3:** Ocular surface evaluation in normal controls and MGD group.

Parameters	Normal controls (n = 114)	MGD group (n = 830)	*P*-value
BCVA	0.9 ± 0.2	0.9 ± 0.2	0.451
IOP (mmHg)	14.0 ± 2.9	13.2 ± 2.5	0.078
OSDI	6.5 ± 4.8	26.0 ± 15.1	< 0.001*
TBUT (s)	7.3 ± 4.2	4.1 ± 2.3	0.002*
Schirmer I test (mm)	11.5 ± 9.4	10.1 ± 8.0	0.250
Lipid layer thickness (nm)	67.9 ± 21.9	51.7 ± 24.9	0.004*
Cornea staining	0.2 ± 0.6	0.7 ± 1.2	0.012*
MG loss (%)	11.5 ± 11.9	34.5 ± 12.2	0.002*
Rate of partial blinking	0.6 ± 0.3	0.6 ± 0.4	0.634
Lid margin abnormality score	1.2 ± 0.7	2.9 ± 0.8	0.002*
MG expressibility score	0.6 ± 0.1	1.7 ± 0.1	0.001*
Meibum score	0.5 ± 0.2	2.2 ± 0.2	0.010*

*BCVA* best-corrected visual acuity, *IOP* intraocular pressure, *MGD* meibomian gland dysfunction, *TBUT* tear film break-up time.

*P < 0.05 was considered statistically significant.

### Depression analysis

In this study, M-SDS was used to evaluate depression and its severity. The score of M-SDS in the MGD group was 30.7 ± 7.7, significantly higher than that in normal controls (27.9 ± 6.2, *P* = 0.001). There were 78 cases (9.4%) with depressive symptoms in the MGD group, which was higher than that in controls (4 cases, 3.5%) (*P* = 0.036). Among MGD cases with depression symptoms, most of them appeared mild (49/78) and moderate (24/78) depressive symptoms. Between normal controls and the MGD group, the severity of depression symptoms was no significant difference (*P* = 0.517, Table 4). Compared with young and middle-aged male or female, older male presented a significant lower SDS value (*P* < 0.001). Similarly, older female exhibited a significant difference of SDS in the comparison with middle-aged female (30, IQR 26–36 vs.28, IQR 23–32.5, *P* = 0.023). There were no significant differences among other subgroups (Supplementary Fig. S1).

**Table 4:** Evaluation of depression symptom in normal controls and MGD group.

Parameters	Normal controls (n = 114)	MGD group (n = 830)	*P* value
Modified self-rating depression scale	27.9 ± 6.2	30.7 ± 7.7	0.001*
Without depression [n (%)]	110 (96.5%)	752 (90.6%)	0.036*
With depression [n (%)]	4 (3.5%)	78 (9.4%)	
Mild depression	4 (3.5%)	49 (5.9%)	0.517
Moderate depression	0 (0)	24 (2.9%)	
Severe depression	0 (0)	5 (0.6%)	

Depression was assessed using the self-rating scale and definite depression was defined as having a depression score of 40 or higher. The severity of depression was rated as mild, 40–50 scores; moderate, 50–60 scores; and severe, above 60 scores.

*P < 0.05 was considered statistically significant.

After analyzing each result of the 17 questions in the M-SDS questionnaire, three parameters (crying spells, sleep disturbance and depressed appetite) had a higher score in the MGD group with depression symptoms, comparing with normal controls with depression (*P* < 0.001, 0.012, 0.008, Table 5).

**Table 5:** Analysis of M-SDS questionnaire survey among depression cases.

Questions	Normal controls (n = 78)	MGD group (n = 4)	*P* value
Depressed affect	1.75 ± 0.96	2.21 ± 0.89	0.322
Diurnal variation	2.25 ± 0.50	2.44 ± 1.09	0.736
Crying spells	1.00 ± 0.00	1.46 ± 0.73	< 0.001*
Sleep disturbance	1.25 ± 0.50	2.68 ± 1.10	0.012*
Depressed appetite	1.25 ± 0.50	2.87 ± 1.19	0.008*
Fear of blindness	2.75 ± 1.26	3.19 ± 1.08	0.431
Weight loss	2.00 ± 0.82	1.58 ± 0.85	0.331
Constipation	1.25 ± 0.50	1.85 ± 1.03	0.257
Tachycardia	1.50 ± 0.58	1.90 ± 0.77	0.311
Fatigue	2.25 ± 1.26	2.56 ± 1.04	0.561
Confusion	3.50 ± 1.00	2.74 ± 1.00	0.144
Psychomotor retardation	2.50 ± 1.29	2.49 ± 1.14	0.983
Irritability	3.00 ± 1.15	2.47 ± 0.88	0.253
Hopelessness	1.75 ± 0.50	2.60 ± 1.00	0.095
Agitation	2.25 ± 1.50	2.54 ± 0.89	0.544
Indecisiveness	2.00 ± 1.41	2.26 ± 1.04	0.636
Personal devaluation	3.00 ± 1.41	2.78 ± 1.03	0.685
Suicidal ideas	3.00 ± 1.15	2.46 ± 0.95	0.276
Dissatisfaction	2.50 ± 1.73	1.67 ± 1.05	0.138
Emptiness	2.50 ± 1.29	2.72 ± 0.99	0.673

*P < 0.05 was considered statistically significant.

In the MGD group, relevant risk factors for depressive symptoms were identified by univariate analysis of odds ratios and were further evaluated by multivariate analysis. The logistic regression analysis showed that the significant univariate odds ratios were 1.95 (1.02, 3.71) for living place, 6.57 (1.08, 39.94) for the history of cerebral infarction, 1.79 (1.08, 2.97) for the history of OSAHS, 3.40 (1.89, 6.12) for the history of allergy, 4.82 (2.39, 9.68) for the history of skin disease, 3.61 (1.48, 8.83) for contact lens wearing, 1.33 (1.07, 1.65) for inactive time per day, 4.76 (1.19, 18.99) for BCVA, and 1.01 (1.00, 1.03) for OSDI. The significant multivariate odds ratios of covariates were as follows: 1.79 (1.00, 3.18) for OSAHS, 2.08 (1.03, 4.22) for allergy, 3.41 (1.48, 7.90) for skin disease, 1.14 (1.04, 1.25) for inactive time, and 4.52 (1.60, 12.75) for contact lens wearing (all *P* < 0.05, Supplementary Table S1).

## Discussion

MGD had a high prevalence worldwide^10–12^. As a leading cause of DED, MGD often presents chronic ocular health problems, especially eye pain, foreign body, and alterations of optical quality, etc. It not only can adversely affect anyone’s life quality but also the mental health of patients. Moreover, depression symptoms also strengthen the subjective feeling about ocular surface discomfort and always coexist with other health conditions. Till now the risk factors and the correlation between MGD and depression remains unclear. Based on our study, the prevalence of depression in the MGD group was higher than normal controls. Risk factors such as OSAHS, allergy, skin disease, contact lens wearing, and inactive time were correlated with the depression symptoms of MGD patients. The results enabled early detection of the signs of mental changes in the MGD group and offer the appropriate support promptly.

In this study, the prevalence of depression in the MGD group was 9.4%, which is significantly higher than that in normal controls (3.5%). As a major cause of DED, MGD can increase the evaporation of tear and decrease the tear volume, which induce short tear breakup times and tear hyperosmolarity. Some studies declared that hyperosmolarity could cause ocular surface inflammation and/or nerve damage, which may increase symptoms by peripheral sensitization. With functional magnetic resonance imaging (fMRI), Yu et al.^13^ found regional homogeneity (ReHo) values of the middle frontal gyrus, inferior frontal gyrus, and superior frontal gyrus were significantly lower in dry eye patients compared to healthy controls. Symptoms of ocular surface injury in DED, especially MGD patients are associated with dysfunction in specific brain regions. Andrianopoulou et al.^14^ investigated brain function and microstructural changes in primary Sjögren syndrome (pSS) and found the functional connectivity abnormalities of the somatosensory cortex and microstructural abnormalities appeared in pSS, which were more pronounced in depression. In addition, depression could also increase the symptom of MGD. Ulusoy et al.^15^ invited 40 newly diagnosed depression patients and found that patients without a history of psychiatric drug use showed dry eye symptoms. Some studies revealed that negative emotional states would induce or enhance the perception of pain and irritation^16^. And the symptoms of pain, dryness, itchiness, stinging, foreign body sensation and sensitivity to light and wind from MGD can also negatively impact the mood of patients and have potential consequences of depression. So, patients with dry eye symptoms but no signs would have the lowest happiness scores. Subjective happiness scores in DED cases were found to be inversely correlated with dry eye symptoms^17^. At last, several studies have confirmed antidepressants may play an important role in provoking DED. Koçer et al.^18^ found that selective serotonin reuptake inhibitors (SSRIs) and serotonin-norepinephrine reuptake inhibitors increased the risk for eye dryness. Furthermore, Zhang et al.^19^ proved that SSRIs could aggravate DED by activating the NF-κB pathway, which shows the interaction between dry eye and depression. Therefore, the depressive symptom could be alleviated by applying the managing MGD, which open the options of cooperative therapy between ophthalmologists and psychiatrists to MGD cases.

Many risk factors make MGD patients more prone to develop depression, for instance, living in rural areas could be associated with depression in our study. However, results from a Korean elder cross-sectional study showed that living in the urban area is also strongly correlated with depression (*P* < 0.001)^3^. The economical differences between developed countries and developing countries may provide a reasonable explanation. Wang et al.^20^ conducted a study about socio-demographic characteristics of depressive symptoms and found that educational attainment, the economic level had a significant association between depressive symptoms, which may explain the different results between Chinese and Korean studies.

Moreover, our study found that some systemic diseases (OSAHS, allergy, skin disease) were also related to depression. The results of the current study are consistent with previous results^21, 22^. A meta-analysis by Jennifer et al. concluded that the risk of developing depressive disorders was two to three times more likely among individuals with two or more chronic conditions^23^. Just as MGD, OSAHS, allergy and skin disease are always chronic and incurable diseases. Chronic pain and irritation are associated with sleep deficiency, activity and mobility limitations, social withdrawal, and loneliness. All these negative effects will induce cognitive impairment, loss of self-confidence and self-esteem, even anxiety and depression^24^.

Wearing a contact lens was the independent risk factor of the development of MGD, especially in rigid contact lens using^25^. And it could also be argued to have potential adverse effects towards the development of depression. Patients with MGD who also wears contact lenses revealed an increase in dry eye symptom and ocular surface staining^26, 27^. Consequently, the shorter TBUT result suggests that MGD and contact lens both contributes to the negative outcome of tear film integrity. Therefore, contact lens may induce depressive symptoms in MGD cases due to the aggravation of dry eye symptoms.

We have applied the M-SDS questionnaire to compare and analyzed some characteristics of depression between the MGD group and controls. For depressive patients in the MGD group, they presented with frequent crying, sleep disturbance and depressed appetite. It is still unclear about the reason causing the repeated crying but trying to get more tear secretion maybe a way to relieve mental tension in patients with MGD. Regarding decreased appetite previous study reported five patients with severe anorexia nervosa, all 5 patients complained of dry and irritated eye symptoms^28^, which indicate a possible cause-effect relationship between dry eye and appetite. Sherwin et al.^29^ revealed that deficiency of vitamin A is a significant risk factor of DED. Change of appetite can potentially decrease the level of intake of vitamin A which then adversely affect depressive symptom. However, no clear evidence articulates the MGD or DED patients' eating habit changes.

Additionally, dry eye and insomnia influence each other in many ways. Ayaki et al.^30^ reviewed 7 articles and concluded that dry eye patients suffer more from bad quality sleep. Wu et al.^31^ also found that poor sleep quality may aggravate DED by affecting tear secretion and tear film stability, even indirectly aggravate depression at the same time. The result from the M-SDS questionnaire showed the difference in frequent crying, sleep disturbance and depressed appetite between MGD patients and control. Another interesting point is that depressive patients in the MGD group may more easily lose hope for their aspirations (2.60 ± 1.00 vs. 1.75 ± 0.50, *P* = 0.095). In Van Leeuwen’s study of patients with Sjögren's syndrome, these patients with a lower “cryability” had a higher score on frustration^32^. Gomes reported that the effect of the quality of life by dry eye may be underestimated^33^.

Our study offered insight into the importance of early detection and intervention of depressive disorder among MGD patients. Questionnaire results regarding sleep, eat changing and cry intention could help ophthalmologists screening depression patients. Such early conversation with the patient can not only prevent further prognosis but also facilitate a speedy recovery.

Even though this study is a focused multi-center cross-sectional study, the sample size could have improved to have more generalizability. Second, alongside the M-SDS questionnaire, other means of measure for the participants mental status could be employed. Third, this study is a cross-sectional study, more robust follow-up evidence is favorable for a deeper understanding of the relationship between MGD and depression.

In conclusion, this study served a pioneering role in connecting MGD and depression. Ophthalmologists should be aware of the association between MGD and depression, better understand patients' mental changes and better treat MGD patients with depression. Furthermore, the correlation between MGD patients with depression and the changing of their cytokines, chemokine, and inflammatory factors could be fascinating to investigate further.

## Methods

### Subjects

A multi-center cross-sectional study was carried out in 23 eye centers with 830 MGD patients and 114 normal controls from outpatient clinics between October 2016 and October 2019 in China. This study was registered in the Chinese Clinical Trial Registry (Registration number: ChiCTR-RDD-17013855) and got ethical approval from the Medical Ethics Committee of Beijing Tongren Hospital (TREC-2016-KY021). All participants were informed of the aims of the study and provided written informed consent was obtained from all subjects according to the declaration of Helsinki.

According to the diagnosis criteria defined by the International Workshop^34^ on MGD in 2011, the inclusion criteria of MGD were as follows: (1) aged ≥ 18 years; (2) presence of at least one subjective symptom, such as ocular fatigue, dryness, foreign body sensation, pain, burning sensation, itching, redness and visual fluctuation; (3) more than one lid margin abnormalities under slit-lamp examination: palpebral margin hyperemia, irregular lid margin, vascular engorgement, plugged meibomian gland orifices, and anterior or posterior replacement of the mucocutaneous junction. Participants with one or more of the following criteria were excluded: (1) eyelid and conjunctival scar; (2) ocular surface abnormalities that may affect the corneal integrity; (3) unwilling or unable to stop medicine that can cause or aggravate dry eye disease; (4) pregnant and lactating women.

The inclusion criteria of the control group were as follows: (1) age ≥ 18 years; (2) no ocular discomfort; (3) no abnormalities of lid margin under slit lamp microscopy. Exclusion criteria included: (1) skin or conjunctival inflammation; (2) eyelid trauma or abnormalities; (3) damaged corneal integrity of any eye; (4) systemic diseases or medications that cause DED; (5) pregnant or lactating women.

### Ocular surface evaluation

**Figure 1:**
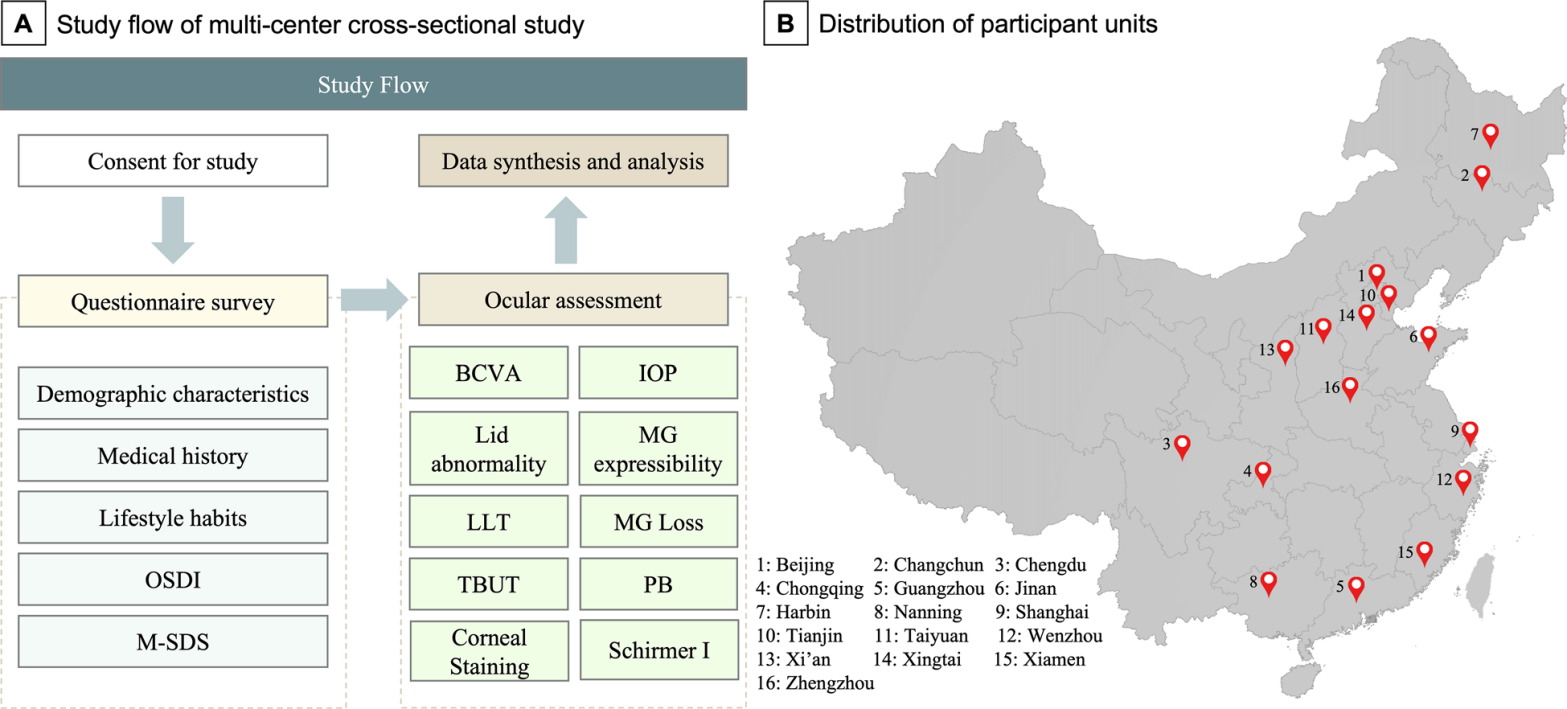
The study flow (Part **A**) and the geographic distribution of 23 eye centers in 16 cities (Part **B**). *OSDI* ocular surface disease index, *M-SDS* modified self-rating depression scale, *BCVA* best corrected visual acuity, *IOP* intraocular pressure, *LLT* lipid layer thickness, *MG* meibomian gland, *TBUT* tear film break-up time, *PB* partial blink. (The figure used with permission from co-author Cao).

Demographic and medical information were collected through a series of questionnaires based on the Beijing Eye Study^35, 36^. These questionnaires included lifestyle queries (reading, sport, walk, etc.), systemic and ocular medical history (hypertension, hyperlipidemia, diabetes mellitus, diabetic retinopathy, glaucoma, cataract, etc.). The smoking index and alcohol drinking index was evaluated by the composite indicators of daily intake (per day) and intake duration (years)^37^. Each subject also underwent a quantification of ocular surface symptoms with the Ocular Surface Disease Index (OSDI) questionnaire (range 0–100). Then, the ophthalmic examinations were performed with the following order: Day 1 finished the best-corrected visual acuity (BCVA), intraocular pressure (IOP), slit-lamp examination (including evaluating lid margin abnormality, meibomian gland expressibility, and meibum scores). Day 2 finished the lipid layer thickness (LLT) measurement, meibomian gland loss evaluation (MGL), partial blink (PB) evaluation, 1 h rest, tear film break-up time (TBUT) measurement, corneal staining, and Schirmer I test (Fig. 1).

BCVA was assessed after automatic refractometry (Auto Refractometer AR-610, Nidek Co., Ltd, Tokyo, Japan). If uncorrected visual acuity was lower than 1.0, subjective refractometry was additionally performed. IOP was measured by pneumotonometry by an ophthalmologist. A slit-lamp examination was carried out by an experienced ophthalmologist and lid abnormalities, meibomian gland dysfunction, corneal disorders were evaluated. Based on the opinion of the international workshop on meibomian gland dysfunction^34^, the lid margin abnormality score was calculated as the presence or absence of the following four parameters: irregularity of the lid margin, vascular engorgement, meibomian gland orifice plugging, and mucocutaneous junction displacement (each presented parameter was given 1 point). Meibum expressibility and the quality of the central five glands in the middle third of the lower lid were evaluated at the slit lamp using the Meibomian Gland Evaluator (TearScience Inc.). Meibum expressibility was scored as: 0 is all five glands expressible and 1 is four, 2 is three, 3 is two, and 4 is 0 glands expressible. Meibum quality was scored as 1, clear; 2, cloudy; 3, cloudy and particulate; and 4, inspissated, and was recorded as the highest grade expressed by the examined glands^38^. The LLT, MGL were measured with the Lipiview II® device (Tear science, Morrisville, NC, USA). With the auto-analysis and calculation of the detection process from the Lipiview II® device, PB was also recorded. The loss ratio of the meibomian glands could be detected after inverted upper and lower lid with infrared imaging technology and ImageJ (National Institutes of Health) software^39^.

TBUT was measured using sterile fluorescein strips impregnated with 0.6 mg fluorescein sodium (Alcon Laboratories, St. Louis, MO, USA). After applying 50 μL of normal saline solution to the paper strip, it was touched to the inferior fornix. The interval between a complete blink and the appearance of the first dry spot was noted. TBUT was measured 3 times and the average was calculated. Corneal staining was recorded by Oxford scale degree under cobalt blue light of silt-lamp^40^. The Schirmer I test was performed without anesthesia, after having the patient's eyes closed for 5 min.

### Depression symptom evaluation

Depression symptoms were evaluated with a questionnaire survey using a modified self-rating depression scale (M-SDS), which was adapted from the Zung self-rated depression scale and had been applied in The Beijing Eye Study^36^. Total scores (range 20–80) were counted by summing the results of each 9 positive questions and 11 negative questions. The 1 to 4 responses to the negative question and positive question using an inverted recording method from 4 to 1. Definite depression was defined as having an M-SDS score of 40 or higher. The severity of depression was rated as mild (40 ~ 50 scores), moderate (50 ~ 60 scores), and severe (above 60 scores). For further analysis, subjects were divided into young (≤ 29 years), middle-aged (30–59), and older (≥ 60 years) groups. Then, six subgroups (young male, young female, middle-aged male, middle-aged female, older male, and older female) were included in this study.

### Data analysis

Statistical analysis was performed with R software (www.r-project.org). For each patient, the right eye was chosen for statistical analysis. Kolmogorov–Smirnov test was used for testing the normality of each variable. Mean values and standardized deviations were used to make the basic statistical description for normally distributed continuous variables, otherwise, median values and interquartile range (IQR) were used. Frequency and percentile were used to make a basic statistical description for categorical variables. An independent two-sample t-test was used to make the comparison of normally distributed continuous variables between the MGD group and control group, while the Wilcoxon rank-sum test was used to make the comparison for non-normally distributed continuous variables. A Chi-square test was used to make the comparison of categorical variables between the MGD group and the control group. Logistic regression analysis was used to explore the association between the M-SDS score, clinical indicators, and depression. Enter approach method was used to calculate univariate odds ratio. Only significant factors in the univariate analysis were used in the multivariate analysis. The significance level was set to be 0.05.

## Supplementary Information

Supplementary Information 1.

Supplementary Information 2.

Supplementary Information 3.

Supplementary Information 4.

Supplementary Information 5.

## Data Availability

All data relevant to the study are included in the article or uploaded as supplemental information.
