# Supplementary figures and images for: A multi-center study evaluating the correlation between meibomian gland dysfunction and depressive symptoms
Source: Sci Rep. 2022 Jan 10;12:443. doi: 10.1038/s41598-021-04167-x (PMC8748897; doi:10.1038/s41598-021-04167-x)

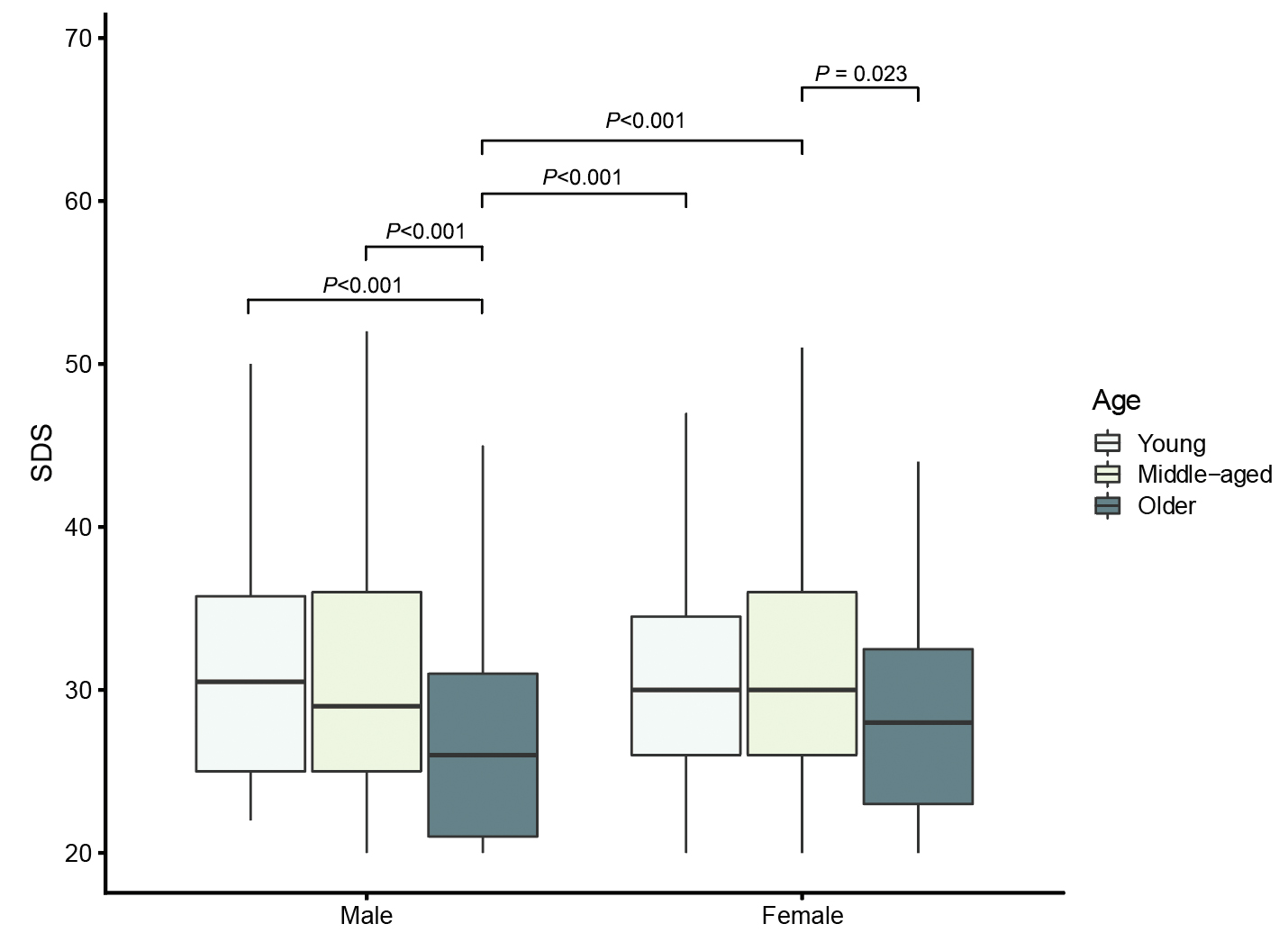

Supplement: Supplementary file 1 — Supplementary Information 1. [file 41598_2021_4167_MOESM1_ESM.jpg]
