# Supplementary material for: A multi-center study evaluating the correlation between meibomian gland dysfunction and depressive symptoms
Source: Sci Rep. 2022 Jan 10;12:443. doi: 10.1038/s41598-021-04167-x (PMC8748897; doi:10.1038/s41598-021-04167-x)
Supplement: Supplementary file 2 — Supplementary Information 2. [file 41598_2021_4167_MOESM2_ESM.docx]

**Supplementary Table 1.** Univariate and multivariate odds ratios in the association between the ocular surface evaluation and depressive symptoms

| **Risk Factors** | **Univariate odds ratio (95% CI)** | ***P* value** | **Multivariate odds ratio (95% CI)** | ***P* value** |
| --- | --- | --- | --- | --- |
| Age | 0.99 (0.97, 1.00) | 0.125 |  |  |
| Gender (Male-Female) | 1.08 (0.66, 1.77) | 0.755 |  |  |
| Living (Urban-Rural) | 1.95 (1.02, 3.71) | 0.042* | 1.29 (0.61, 2.72) | 0.508 |
| Hypertension | 0.54 (0.19, 1.51) | 0.237 |  |  |
| Hyperlipidemia | 0.39 (0.12, 1.27) | 0.118 |  |  |
| Diabetes mellitus | 0.83 (0.19, 3.61) | 0.808 |  |  |
| Coronary heart disease | 2.80 (0.57, 13.73) | 0.204 |  |  |
| Cerebral infarction | 6.57 (1.08, 39.94) | 0.041* | 7.83 (0.97, 63.41) | 0.054 |
| OSAHS | 1.79 (1.08, 2.97) | 0.025* | 1.79 (1.00, 3.18) | 0.049* |
| Allergy | 3.40 (1.89, 6.12) | <0.001* | 2.08 (1.03, 4.22) | 0.042* |
| Skin disease | 4.82 (2.39, 9.68) | <0.001* | 3.41 (1.48, 7.90) | 0.004* |
| Ametropia | 1.06 (0.66, 1.71) | 0.806 |  |  |
| Contact lens | 3.61 (1.48, 8.83) | 0.005* | 4.52 (1.60, 12.75) | 0.004* |
| Diabetic retinopathy | 0.83 (0.21, 3.68) | 0.816 |  |  |
| Glaucoma | 1.95 (0.19,1.51) | 0.052 |  |  |
| Cataract | 0.87 (0.30, 2.49) | 0.795 |  |  |
| Smoking | 1.03 (0.99, 1.07) | 0.146 |  |  |
| Drinking | 1.00 (1.00, 1.01) | 0.625 |  |  |
| Sport | 1.43 (1.10, 1.85) | 0.052 | 1.19 (0.90, 1.58) | 0.214 |
| Reading |  |  |  |  |
| 0 (never) | 0.83 (0.21, 3.68) | 0.816 |  |  |
| 1 (almost never) | 1.33 (0.41, 4.32) | 0.632 |  |  |
| 2 (1-2day/week) | 1.01 (0.30, 3.44) | 0.983 |  |  |
| 3 (3-5day/week) | 0.76 (0.22, 2.55) | 0.651 |  |  |
| 4 (almost every day) | 0.55 (0.18, 1.64) | 0.281 |  |  |
| Inactive time | 1.33 (1.07, 1.65) | 0.011* | 1.14 (1.04, 1.25) | 0.006* |
| BCVA | 4.76 (1.19, 18.99) | 0.027* | 1.17 (0.60, 2.29) | 0.640 |
| IOP | 1.07 (0.98, 1.17) | 0.147 |  |  |
| OSDI | 1.02 (1.00, 1.03) | 0.012* | 1.00 (0.98, 1.01) | 0.632 |
| TBUT | 0.89 (0.79, 1.00) | 0.052 |  |  |
| Schirmer I | 1.00 (0.97, 1.03) | 0.995 |  |  |
| Lipid layer thickness | 1.00 (1.00, 1.01) | 0.355 |  |  |
| Cornea staining | 1.00 (0.82, 1.22) | 0.982 |  |  |
| MG loss | 0.98 (0.96, 1.01) | 0.134 |  |  |
| Rate of partial blinking | 0.72 (0.38, 1.38) | 0.326 |  |  |
| Lid margin abnormality score | 1.36 (0.73, 2.53) | 0.336 |  |  |
| MG expressibility score | 0.99 (0.58, 1.68) | 0.962 |  |  |
| Meibum score | 0.76 (0.10, 5.82) | 0.789 |  |  |

Note: **P*<0.05 was considered statistically signiﬁcant.
