# Supplementary material for: A multi-center study evaluating the correlation between meibomian gland dysfunction and depressive symptoms
Source: Sci Rep. 2022 Jan 10;12:443. doi: 10.1038/s41598-021-04167-x (PMC8748897; doi:10.1038/s41598-021-04167-x)
Supplement: Supplementary file 3 — Supplementary Information 3. [file 41598_2021_4167_MOESM3_ESM.docx]

**Supplementary Table 2:** Detailed analysis of M-SDS score in MGD subgroups

| **Parameters** | **M-SDS score** | ***P* value** | **With depression [n (%)]** | ***P* value** |
| --- | --- | --- | --- | --- |
| **Age** |  |  |  |  |
| ≤29 (n=173) | 31.4±8.0† | <0.001* | 20 (11.6%) | 0.417 |
| 30-59 (n=555) | 31.0±7.7† |  | 51 (9.2%) |  |
| ≥60 (n=102) | 28.1±6.7 |  | 7 (6.9%) |  |
| **Gender** |  |  |  |  |
| Male (n=290) | 30.2±7.6 | 0.175 | 26 (9.0%) | 0.754 |
| Female (n=540) | 31.0±7.7 |  | 52 (9.6%) |  |

Note: **P*<0.05 was considered statistically signiﬁcant. † means the significant difference between this subgroup and the “age ≥60” subgroup.
