# Supplementary material for: A multi-center study evaluating the correlation between meibomian gland dysfunction and depressive symptoms
Source: Sci Rep. 2022 Jan 10;12:443. doi: 10.1038/s41598-021-04167-x (PMC8748897; doi:10.1038/s41598-021-04167-x)
Supplement: Supplementary file 4 — Supplementary Information 4. [file 41598_2021_4167_MOESM4_ESM.docx]

| **Parameters** | **Test** |  | **Parameters** | **Test** |
| --- | --- | --- | --- | --- |
| Age | Normal |  | OSDI | Normal |
| Gender | Normal |  | TBUT | Normal |
| Residence | Non-normal |  | Schirmer I test | Normal |
| Systemic diseases | Non-normal |  | Lipid layer thickness | Normal |
| Ocular diseases | Non-normal |  | Cornea staining | Normal |
| Smoking index of smokers | Normal |  | MG loss | Normal |
| Alcohol drinking index of drinkers | Normal |  | Rate of partial blinking | Normal |
| Other parameters of life habits | Non-normal |  | Lid margin abnormality score | Normal |
| Modified self-rating depression scale | Normal |  | MG expressibility score | Normal |
| Proportion of depression | Non-normal |  | Meibum score | Normal |
| Question analysis of M-SDS | Normal |  | Univariate odds ratio | Non-normal |
| BCVA | Normal |  | Multivariate odds ratio | Non-normal |
| IOP | Normal |  |  |  |

**Supplementary Table 3:** Normal and non-normal distribution situation of parameters
