# Supplementary material for: A multi-center study evaluating the correlation between meibomian gland dysfunction and depressive symptoms
Source: Sci Rep. 2022 Jan 10;12:443. doi: 10.1038/s41598-021-04167-x (PMC8748897; doi:10.1038/s41598-021-04167-x)
Supplement: Supplementary file 5 — Supplementary Information 5. [file 41598_2021_4167_MOESM5_ESM.docx]

**Supplementary Figure 1.** Comparison of SDS values in the different subgroups (young ≤ 29 years, middle-aged: 30-59 years and older ≥ 60 years).
